# Regional discrepancies in the microphysical attributes of summer season rainfall over Taiwan using GPM DPR

**DOI:** 10.1038/s41598-023-38245-z

**Published:** 2023-07-26

**Authors:** Jayalakshmi Janapati, Balaji Kumar Seela, Pay-Liam Lin

**Affiliations:** 1 Institute of Atmospheric Physics, Department of Atmospheric Sciences, National Central University, Zhongli District, Taoyuan City, 320317 Taiwan; 2 Earthquake-Disaster and Risk Evaluation and Management Center, National Central University, Zhongli District, Taoyuan City, 320317 Taiwan; 3 Research Center for Hazard Mitigation and Prevention, National Central University, Zhongli District, Taoyuan City, 320317 Taiwan

**Keywords:** Climate sciences, Atmospheric science

## Abstract

Taiwan, an island located in the northwest Pacific region, is influenced by heavy rainfall events during warm seasons, particularly from June to August. Interaction of precipitating clouds with the complex topography results in inhomogeneous and intense rainfall over Taiwan. Hence, the present study investigates the regional discrepancies in the microphysical characteristics of summer season rainfall over (north, south, east, and central) Taiwan using 9 years (2014–2022) of GPM DPR measurements. The results showed clear distinctions in the precipitation and raindrop size distributions over the north, south, east, and central Taiwan. The contoured frequency by altitude diagrams (CFADs) of radar reflectivity, rainfall rate, drop diameter, and concentration clearly infer the dominance of large-size super cooled liquid and ice particles above the melting layer and rain particles below the melting layers in the south and central Taiwan. Central (north) Taiwan is dominated by large-size (small) drops among four regions. Higher concentrations of large drops over central Taiwan (principally from convective precipitation) and south Taiwan (primarily from stratiform precipitation) is attributed to higher rainfall amounts over these two regions than the north and east Taiwan. Furthermore, irrespective of precipitation type and geographic region, summer monsoon rainfall over Taiwan is dominated by coalescence and breakup processes. The microphysical characteristics of summer season rainfall addressed in this study could assist in refining the cloud modeling simulations over complex topography in Taiwan.

## Introduction

Taiwan Island, located in the East Asia region, is primarily influenced by southwest and northeast monsoon flows. Based on atmospheric stability and prevailing winds, two monsoon flows over Taiwan are classified into six seasons (winter, spring, mei-yu, summer, typhoon and autumn)^1^. Among these six seasons, the summer (16 June to 31 August) is primarily influenced by the convective systems embedded with southwesterly monsoon flow^2–4^. The interactions of summer season convective systems with the complex topography of Taiwan brings heavy rainfall throughout this island, resulting in devastating socio-economic consequences like flash floods, debris flow, landslides, and crop damage^5–8^. Such extreme disasters from intense rainfall enforced mounting attention on dynamical and thermo-dynamical characteristics of summer season rainfall over this island^1–3,8–10^.

Mitigation and assessment of heavy rainfall events dictate to progress the precipitation estimation algorithms and cloud modeling simulations based on the enhanced understanding of cloud and rain microphysical process^11–16^. Such goals in Taiwan can be achieved with the aid of precipitation measurement instruments like disdrometers and radars. Hence, using ground-based disdrometers and radars, there have been reports on the microphysical attributes of summer season rainfall over Taiwan. For instance, inter comparison of summer season raindrop size distribution (RSD) measurements with disdrometers from the north Taiwan and Palau islands demonstrated a higher concentration of large drops in Taiwan than in Palau. Such distinctions were caused by the disparities in the amount of aerosol loading and convection over these two islands^17^. Besides, investigations on the raindrop size distribution measurements of summer and winter rainfall over north Taiwan, using a ground-based disdrometer, by Seela et al.^18^ revealed bigger size drops in summer than in the winter season. They attributed the RSD differences to the association of intense and deep storms in summer and shallow clouds in winter. Using the same disdrometer measurements and radar reflectivity profiles from six ground-based radars over north Taiwan, Lee, et al.^19^ investigated the rain and cloud microphysical properties of six (winter, spring, mei-yu, summer, autumn, and typhoon) seasons. They perceived high (low) vertical structures in summer, mei-yu, and typhoons (winter, spring, and autumn), with more large size drops in summer and smaller size drops in winter. In addition, the inequalities in the raindrops size distributions of summer season rainfall, segregated into typhoon and non-typhoon rain, are also reported over north Taiwan^20^. In accordance with the findings of Lee, et al.^19^, based on three ground-based disdrometers’ measurements from north Taiwan, Janapati, et al.^21^ also revealed higher mass-weighted mean diameter and rainfall kinetic energy values in Mei-yu, summer and typhoon seasons than the rest three (winter, spring, and autumn) seasons.

Apart from the mentioned above disdrometer studies over Taiwan, cloud/rain microphysics of different precipitations were also reported with ground-based radars^22–31^. As an example, with the aid of ground-based polarimetric radar measurements from south Taiwan SoWMEX/TiMREX (Southwest Monsoon Experiment/Terrain-Influenced Monsoon Rainfall Experiment) field campaign, Gao, et al.^29^ tried to improve the two-moment cloud microphysics schemes bulk microphysics scheme of the Chinese Academy of Meteorological Sciences and Weather Research and Forecasting model. Moreover, using the same SoWMEX/TiMREX polarimetric radar measurements, Chang, et al.^28^ investigated the precipitation efficiency and associated microphysical and kinematic characteristics of a squall line and mesoscale convective system. They hypothesized that the enhanced precipitation efficiency observed in the mesoscale convective system was ascribed to the enriched accretion growth of rain at the lower levels embedded with surplus water content. Recently, the microphysical processes of severe wintertime rainfall over Yilan county in East Taiwan were analyzed using ground-based radar during the YESR2020 (Yilan Experiment of Severe Rainfall 2020) field campaign conducted in November 2020^30^.

Even though there were adequate microphysical studies in Taiwan, they were limited to case studies or to a specific region of Taiwan. In addition, owing to the scarcity of ground-based measurements over complex topographic areas of Taiwan, comprehensive investigations on the precipitation microphysical processes over the complete island are yet to be documented. Such measurement inhibits can be overcome with remote sensing instruments like global precipitation measurement mission dual-frequency precipitation radar (GPM DPR) which can provide the estimated particle size distribution information globally. Henceforth, the present study is intended to investigate the discrepancies in the rainfall and microphysical characteristics of the summer season (16 June–31 August) rainfall over four (north, central, east, and south) regions of Taiwan. More specifically, the key questions heighten in this study are: 1. Are there any disparities in the spatial distribution of summer rainfall over Taiwan? 2. What is the role of Taiwan terrains (central mountain ranges) on rainfall distribution, 3. What are the microphysical processes responsible for the regional variations in summer rainfall? With this brief introduction, the data sets used in the present study are mentioned in “Study area and data” Section, results and discussion are detailed in “Results and discussion” Section, followed by the summary of the present study in “Summary” Section.

## Study area and data

**Figure 1:**
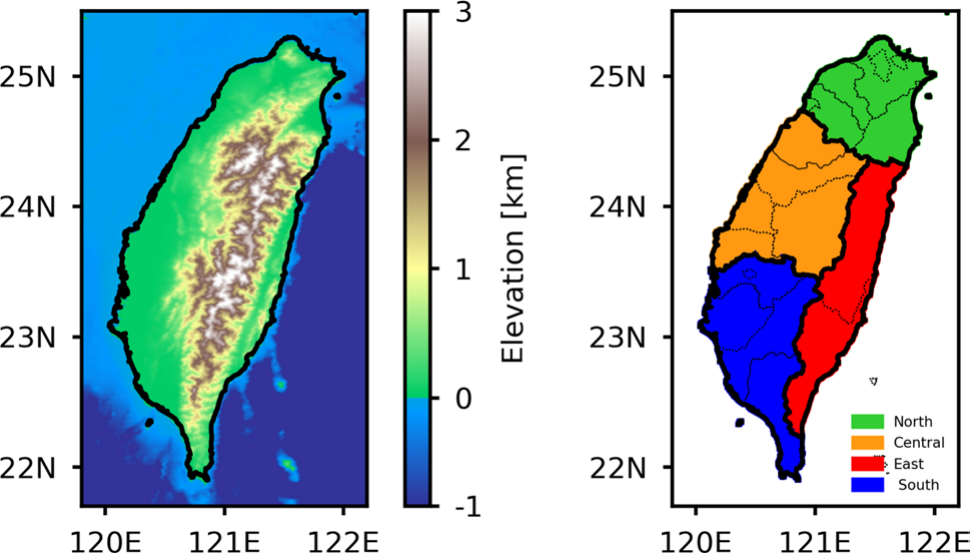
Taiwan (**a**) topographic map with (**b**) north, central, east, and south regions in different color shades. The Taiwan topographic map is generated using publicly archieved Python code in the github repository (available at https://github.com/13ff6/Topography_Map_Madagascar).

Taiwan, a mountainous subtropical island in the western Pacific, has a complex topography, which includes central mountain ranges (CMR) extending from southwest to northeast of the island. The CMR of Taiwan acts as a natural topographic barrier to the monsoon flows, providing an excellent opportunity to understand the interaction between the monsoon convective systems and the topography. The topographic map of Taiwan with four geographic regions is demonstrated in Fig. 1. While analyzing the spatial distribution of summer season rainfall, the Taiwan geographic map is classified into north, central, south, and east regions^32^, and are depicted with green, orange, blue, and red-filled areas in Fig. 1.

After the successful completion of tropical rainfall measurement mission (TRMM), the GPM, a joint mission between the USA and Japan, was launched in 2014 to estimate the precipitation (rain and snow) with an extended global coverage (65°S and 65°N) than the TRMM^33–35^. The GPM DPR estimates the precipitation (surface to an altitude of 22 km) at the nadir with a spatial and vertical resolution of 5 km and 125 m, respectively. The dual-frequency precipitation radar of GPM operating at Ka- and Ku-band frequency can provide the retrieved rainfall and precipitating particles size information like mass-weighted mean diameter (*D*_*m*_, mm) and normalized intercept parameter (dB*N*_*w*_ = 10log_10_*N*_*w*_, *N*_*w*_ in m^−3^ mm^−1^) by using single- or dual-frequency algorithm^36–38^. While retrieving the RSD parameters in the single- or dual-frequency algorithms, the precipitating particles are assumed to exhibit the normalized gamma distribution with constant shape parameter (*μ* = 3)^39^. Along with the rain and particle size information, the level 2 data products of GPM DPR deliver the precipitation type (stratiform, convective, and other) information, which is executed in the classification module of the GPM DPR (either by single- or dual-frequency) precipitation estimation algorithm. In the single frequency method, the precipitation is segregated into stratiform, convective or other type using vertical profile method (V method) and horizontal pattern methods (H method)^40–42^. In the case of dual frequency methods, the vertical profile information of differential reflectivity ration (DFR_m_) is used to classify the precipitation into stratiform, convective, and other type. Owing to the significant influence of non-Rayleigh scattering and the differential path integrated attenuation in the melting layer, the vertical profile of DFR_m_ can serve in identifying the melting layer and precipitation type^43–45^. In the classification module (CFS) of the 2ADPR (GPM DPR Preciptation Profile L2A 1.5 h 5 km V07), reliable bright band detection is confirmed using combined information of bright band detection by DRF_m_ and Ku-only algorithm methods. To indulge the microphysical attributes of summer season rainfall over Taiwan, the present study utilized the near-surface and vertical profiles of rainfall rate (*R*, mm h^−1^), radar reflectivity (*Z*, dBZ), *D*_*m*_ (mm), dB*N*_*w*_ (*N*_*w*_ in m^−3^ mm^−1^) from the level 2 data product of GPM DPR (version 7).

## Results and discussion

### Spatial distributions of near-surface rain and RSD parameters

**Figure 2:**
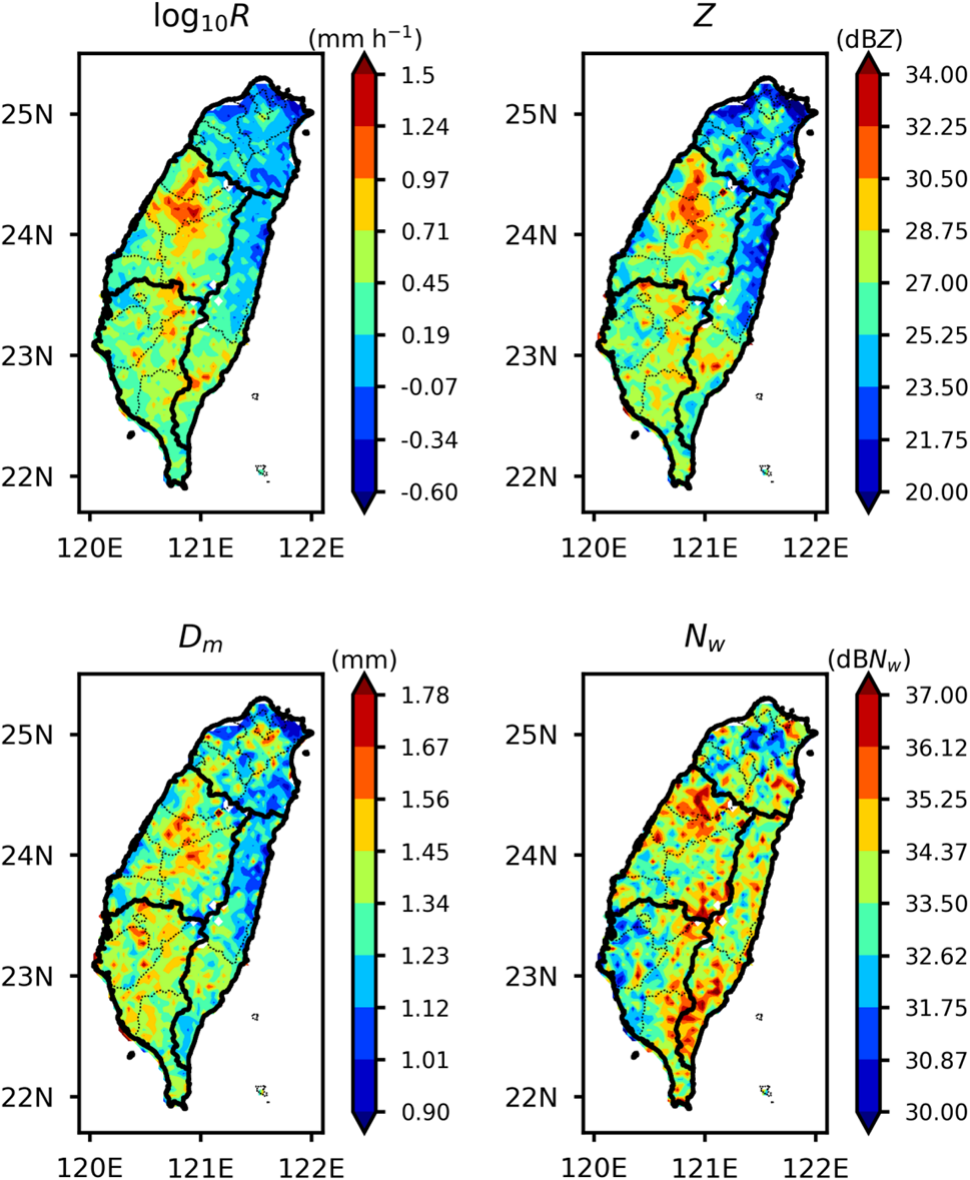
Spatial distribution of near-surface (2 km) (**a**) precipitation rate (*R*, mm h^−1^), (**b**) radar reflectivity (*Z*, dBZ), (**c**) mass-weighted mean diameter (*D*_*m*_, mm), (**d**) normalized intercept parameter (dB*N*_*w*_:10log_10_*N*_*w*_, *N*_*w*_ in m^−3^ mm^−1^) for the summer season rainfall over Taiwan.

Spatial distributions of mean rainfall rate (*R*, mm h^−1^), radar reflectivity (*Z*, dBZ), *D*_*m*_ (mm), dB*N*_*w*_ (*N*_*w*_ in m^−3^ mm^−1^) for the summer season rainfall over Taiwan are illustrated in Fig. 2. Distributions of rainfall rate and raindrop size distribution (RSD) parameters (*Z*, *D*_*m*_, and dB*N*_*w*_) apparently indicate disparities among four (north, central, east, and south) regions of Taiwan. Very low mean rainfall intensities (< 0.1 mm h^−1^) are observed over the northern tip of Taiwan. Central and south regions are associated with relatively higher rainfall rates than the north and east part of Taiwan. Central Taiwan is dominated by higher rainfall intensities than the other three regions. Such distinctions in the spatial distribution of rainfall over Taiwan, with pronounced rainfall intensities in central and south Taiwan, were also documented in previous studies^1,2,8^. The intense rainfall over south and central Taiwan can be attributed to the interaction of the southwesterly monsoon flow convective systems with the central mountain ranges^8^. In accordance with the rainfall rate, the radar reflectivity and *D*_*m*_ values are higher over central and south Taiwan than the north and east Taiwan. However, the central and eastern regions of Taiwan show relatively higher number concentration than the north and south region. It is worth noting that the higher concentration of large drops (larger *D*_*m*_ and dB*N*_*w*_ values) contributes to higher rainfall rates in central Taiwan. On the other hand, eastern Taiwan is dominated by a larger concentration of *D*_*m*_ values of less than 1.24 mm.

### Near-surface RSD characteristics

**Figure 3:**
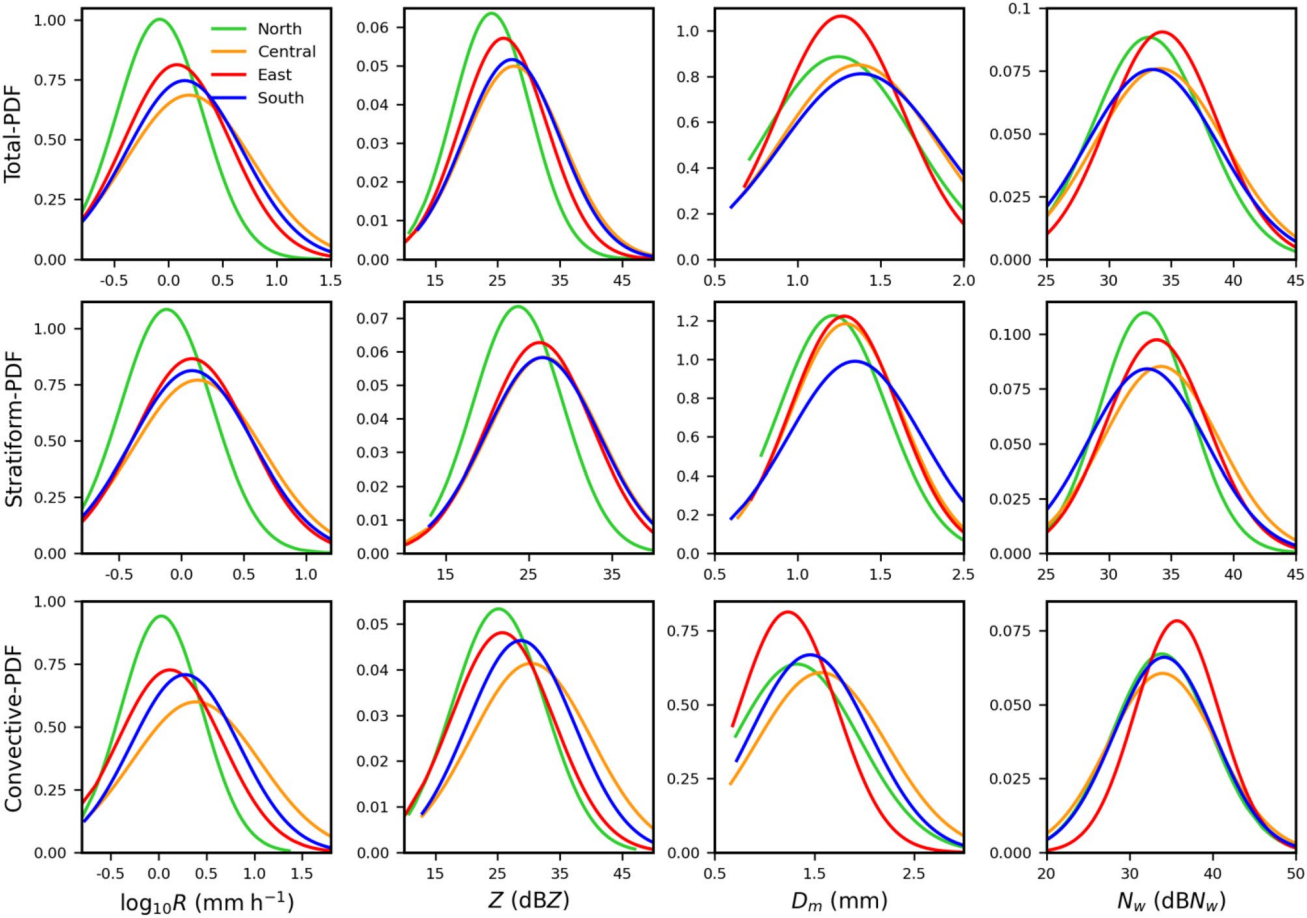
Probability density function (PDF) of near-surface (2 km) rainfall rate (*R*, mm h^−1^) (first column), radar reflectivity (*Z*, dBZ) (second column), mass-weighted mean diameter (*D*_*m*_, mm) (third column), and normalized intercept parameter (dB*N*_*w*_: 10log_*10*_*N*_*w*_, *N*_*w*_ in m^−3^ mm^−1^) (fourth column) for the summer season rainfall over north, central, east and south Taiwan. First, second, and third row corresponds to total, stratiform, and convective precipitations, respectively.

The probability density functions (PDF) of the near-surface (2 km) rainfall rate (*R*, mm h^−1^), radar reflectivity (*Z*, dBZ), *D*_*m*_ (mm), and dB*N*_*w*_ (*N*_*w*_ in m^−3^ mm^−1^) are illustrated in Fig. 3 to elucidate the distinctions in the RSD parameters in four regions of Taiwan. The percentage of stratiform (convective) precipitation in north, central, east, and south regions of Taiwan are around 66% (34%), 71% (29%), 74% (26%), 60% (40%), respectively. The PDF plots demonstrated a higher occurrence percentage of rainfall rates, especially for log_10_*R* > 0.6, in central and south than the north and east regions for total, stratiform, and convective precipitations. More particularly, the distinctions in the PDF distribution of rainfall rate (log_10_*R* > 0.6) are more predominant in the convective precipitation, with a lower (higher) occurrence percentage in north (central) Taiwan. The total, stratiform, and convective precipitations of north Taiwan exhibit higher occurrence frequency at lower rainfall rates (log_10_*R* < 0.25) and lower occurrence frequency at higher rainfall rates (log_10_*R* > 0.5) in stratiform and convective precipitations. In addition to that, excluding north Taiwan, stratiform precipitations of the rest three regions divulge nearly identical occurrence frequency at higher rainfall rates (log_10_*R* > 0.5). For the total and convective rainfall, central and south Taiwan show higher occurrence frequency for the radar reflectivity > 30 dBZ, followed by east and north Taiwan. The central and south Taiwan stratiform precipitations hint a higher occurrence frequency at Z > 30 dBZ. Following the radar reflectivity, the mass-weighted mean diameter of south and central Taiwan exhibits higher PDF frequency at *D*_*m*_ values > 1.5 mm than the east and north Taiwan. Interestingly, for *D*_*m*_ > 1.5 mm, south Taiwan shows a higher occurrence than central Taiwan in stratiform precipitation; however, an opposite characteristic can be seen in the case of convective rainfall with a higher occurrence frequency in central than south Taiwan. The normalized intercept parameter frequency distribution doesn’t reveal distinguishable differences among the four regions. Despite that, north Taiwan shows a lower percentage at higher dB*N*_*w*_ values (dB*N*_*w*_ > 35) in stratiform precipitation. The convective precipitation of east Taiwan shows a higher occurrence frequency of number concentration for dB*N*_*w*_ > 35. Such a higher percentage of dB*N*_*w*_ in the convective precipitation of east Taiwan is due to the low density of the relatively large drops over 2 mm. From the PDF distributions of RSD parameters, it can be inferred that the four regions of Taiwan specify substantial differences in the RSD parameters (log_10_*R*, *Z*, and *D*_*m*_), predominantly in the convective precipitation.

**Figure 4:**
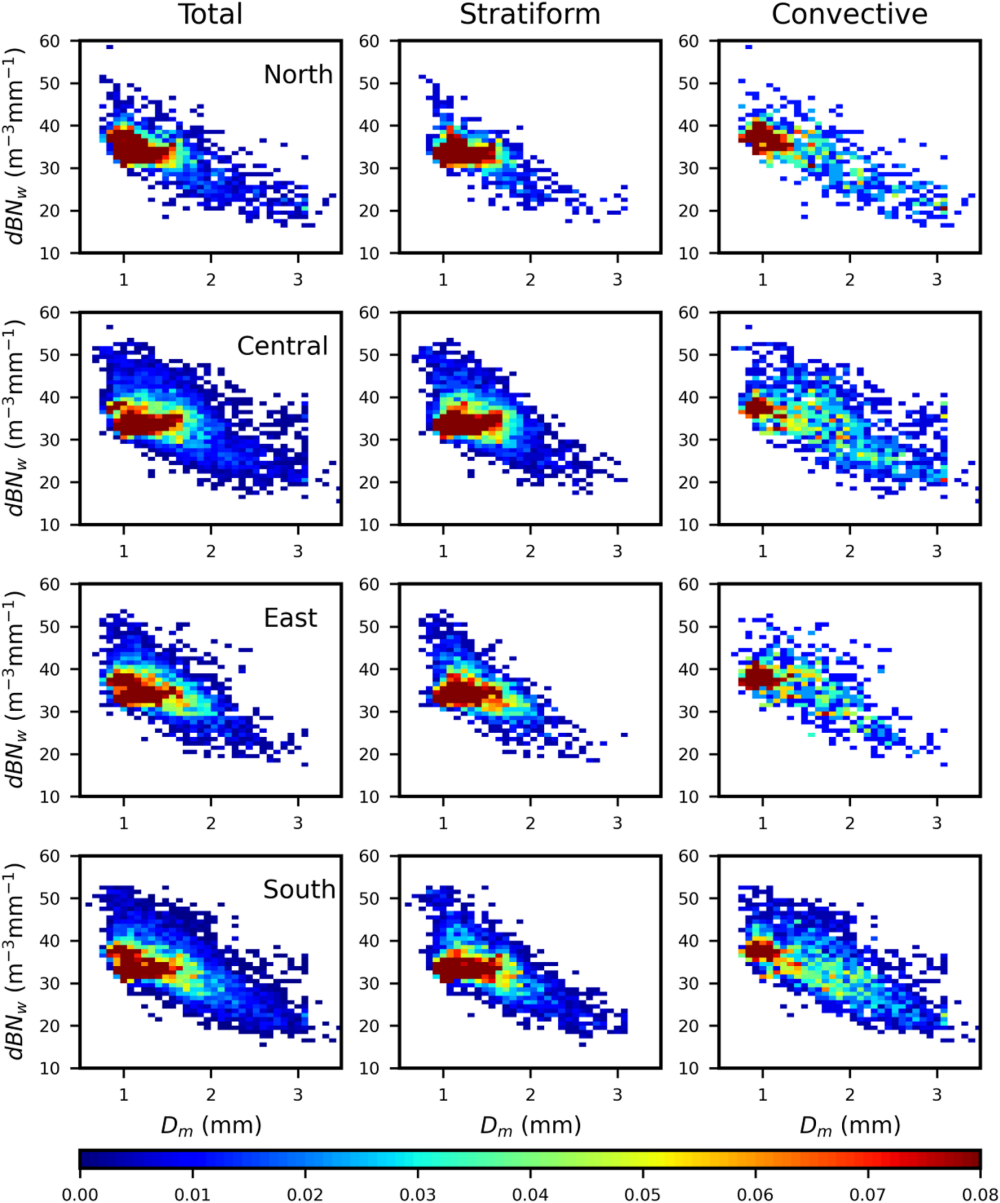
Two dimensional histogram between the near-surface (2 km) mass-weighted mean diameter (*D*_*m*_, mm) and normalized intercept parameter (dB*N*_*w*_: 10log_*10*_*N*_*w*_, *N*_*w*_ in m^−3^ mm^−1^) for summer season rainfall over north (first row), east (second row), central (third row) and south (fourth row) Taiwan for total (first column), stratiform (second column), convective (third column).

The occurrence frequency distribution of mass-weighted mean diameter (*D*_*m*_) and normalized intercept parameters (dB*N*_*w*_) for total, stratiform, and convective precipitations are estimated, as depicted in Fig. 4, to reveal the near-surface RSD characteristics in four regions of Taiwan. Regardless of the precipitation (total/stratiform/convective) type, the drop concentration (dB*N*_*w*_) decreases with the increase in size (*D*_*m*_) at four regions of Taiwan. The mean *D*_*m*_ (*N*_*w*_) values for the total precipitation of north, central, east, and south Taiwan are, 1.24, 1.36, 1.26, 1.38 mm (33.21, 34.10, 34.29, 33.48 dB*N*_*w*_), respectively. The average *D*_*m*_ (*N*_*w*_) values for the stratiform precipitations of north, central, east, and south Taiwan are, respectively, 1.21, 1.29, 1.28, 1.34 mm (32.89, 34.18, 33.84, 33.07 dB*N*_*w*_). Furthermore, the mean *D*_*m*_ (*N*_*w*_) values for north, central, east, and south Taiwan are 1.33, 1.57, 1.23, 1.46 mm (33.87, 33.97, 35.70, 34.15 dB*N*_*w*_), respectively.The peak occurrence frequency of dB*N*_*w*_ and *D*_*m*_ ranges between 30 − 40 and 0.5 − 1.5 mm, respectively, for the total precipitation of four regions. Nonetheless, stratiform and convective precipitations of the four regions display distinct characteristics. For instance, north Taiwan stratiform (convective) precipitation indicates higher concentration (dB*N*_*w*_ between ~ 30 − 40) for the raindrops of diameter ~ 1 − 1.7 mm (~ 1 − 1.4 mm). Similar to north Taiwan, rest three regions also demonstrate a higher concentration (dB*N*_*w*_ between ~ 30 − 40) of raindrops of diameter below 1.8 mm (1.3 mm) in stratiform (convective) precipitation. This clearly indicates the dominant concentration of small and mid-size drops (*D*_*m*_ < 1.8 mm) in stratiform precipitation and small-size drops (*D*_*m*_ ≤ 1.2 mm) in convective precipitation. In contrast, in the stratiform precipitation, excluding north Taiwan, the rest three regions demonstrated nearly identical concentrations for the raindrops of *D*_*m*_ ranging between 1 − 1.8 mm. On the contrary, in the convective precipitation, the central and south Taiwan point out higher concentrations for the raindrops of *D*_*m*_ ranging from 1 − 2.8 mm than the north and east Taiwan. The unavailability of higher concentrations of large drops in north Taiwan and the higher concentration of large drops in south and central Taiwan leads to smaller (higher) rainfall rates in north (south and central) Taiwan.

### Vertical distributions of rain and RSD parameters

**Figure 5:**
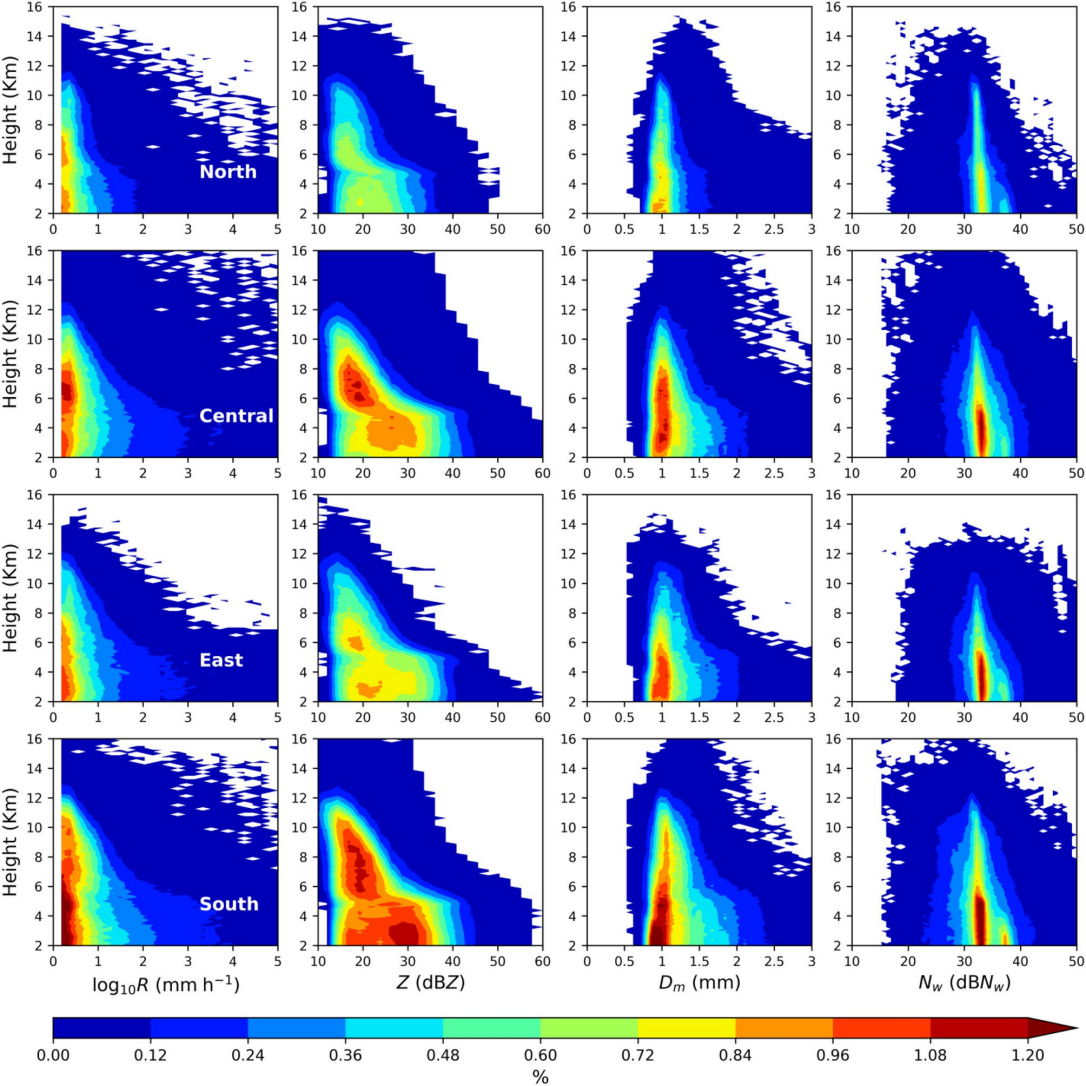
Contour frequency by altitude diameter (CFAD) of rainfall rate (*R*, mm h^−1^) (first column), radar reflectivity (*Z*, dBZ) (second column), mass-weighted mean diameter (*D*_*m*_, mm) (third column), and normalized intercept parameter (dB*N*_*w*_: 10log_*10*_*N*_*w*_, *N*_*w*_ in m^−3^ mm^−1^) (fourth column) for the summer season *total rainfall* over north (first row), central (second row), east (third row) and south (fourth row) Taiwan.

As depicted in Fig. 5, the vertical characteristics of RSD parameters over the four regions of Taiwan are understood using the contour frequency by altitude diameter (CFAD) of rainfall rate (*R*, mm h^−1^), radar reflectivity (*Z*, dBZ), *D*_*m*_ (mm), and dB*N*_*w*_ (*N*_*w*_ in m^−3^ mm^−1^)^46^. The rainfall rate CFADs of the total precipitation implies remarkable variations among the four regions with higher occurrence percentage of higher rainfall rates (log_10_*R* > 1) over south and central Taiwan. Among the four regions, north Taiwan shows a substantially smaller rainfall rate, followed by east Taiwan. The CFADs of radar reflectivity show a predominant occurrence percentage in the south, followed by central and east Taiwan. The extent of 30 dBZ reflectivity is deeper in the southern region than in the remaining three regions. It is worth noting that a higher occurrence frequency of radar reflectivity can be seen in the cold rain regions (> 5 km) of the south Taiwan precipitating clouds, followed in central Taiwan; however, such a higher occurrence percentage is feeble in the east region and mostly absent in the north region. Additionally, in the warm rain regions (< 5 km), radar reflectivity values ranging from 18 − 33 dBZ, 15 − 38 dBZ, and 18 − 31 dBZ denote higher (> 0.72%) occurrence percentages in central, south, and east Taiwan, respectively; nonetheless, such higher occurrence percentage of radar reflectivity are absent over north Taiwan. The CFADs of *D*_*m*_ show a higher occurrence frequency of around 1 mm in central, east, and south Taiwan; conversely, the extent of the higher occurrence frequency is deeper (> 6 km) in south and central Taiwan. Below the melting layer, the drops of diameter greater than 1.5 mm show dominant occurrence in south and central Taiwan. Among four regions, excluding north Taiwan, the other three regions express nearly identical normalized intercept parameter frequency distributions. The higher frequency of dB*N*_*w*_ (about 38) in the lower levels of south Taiwan can be related to smaller drops (Dm < 1 mm) produced by the collision-breakup processes in the convective precipitation.

**Figure 6:**
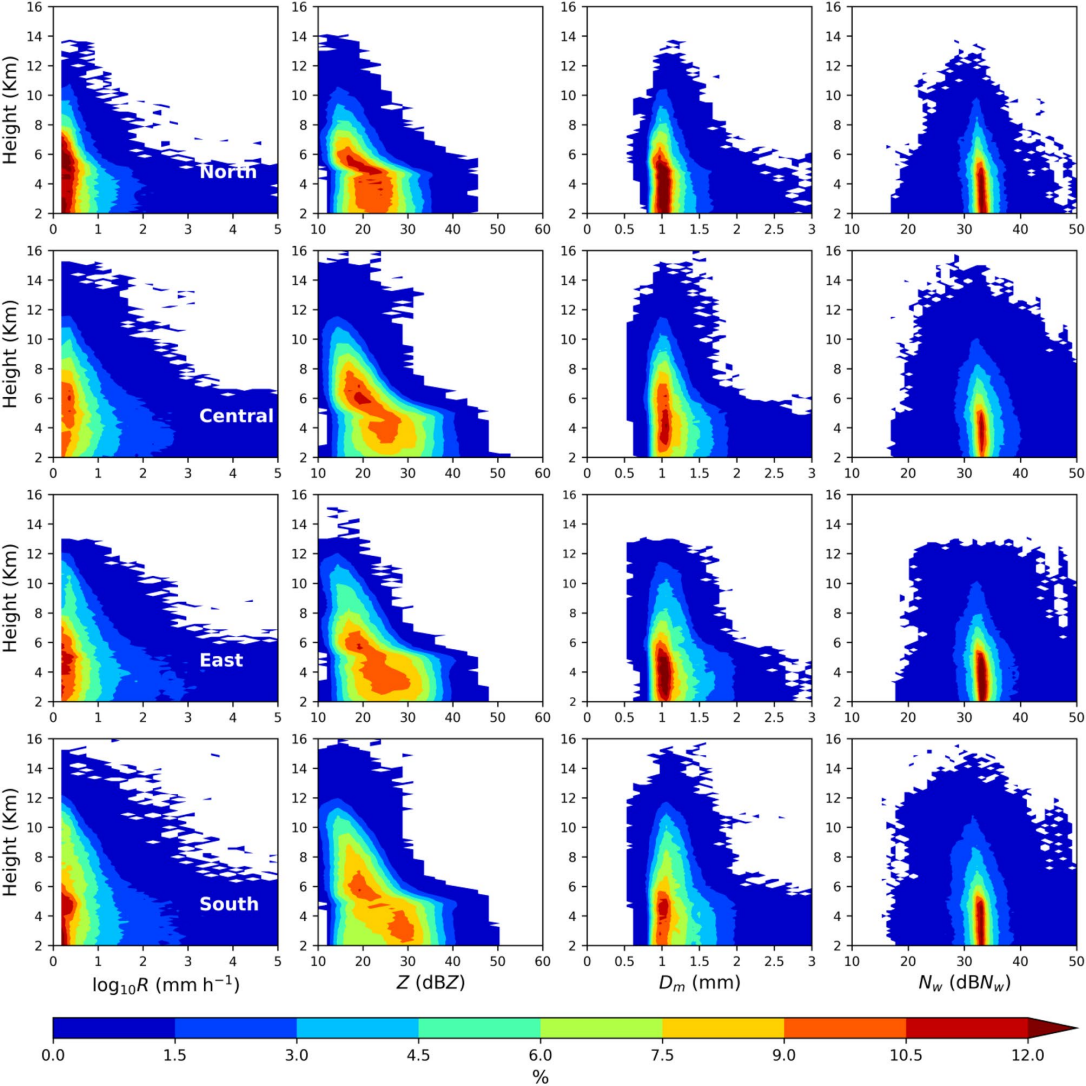
Contour frequency by altitude diameter (CFAD) of rainfall rate (*R*, mm h^−1^) (first column), radar reflectivity (*Z*, dBZ) (second column), mass-weighted mean diameter (*D*_*m*_, mm) (third column), and normalized intercept parameter (dB*N*_*w*_: 10log_*10*_*N*_*w*_, *N*_*w*_ in m^−3^ mm^−1^) (fourth column) for the summer season *stratiform rainfall* over north (first row), central (second row), east (third row) and south (fourth row) Taiwan.

As given in Fig. 6 for the stratiform precipitation, among the four regions’ rainfall rates CFADs, north Taiwan has a higher frequency of lower rainfall rates with deeper extent (above 5 km) than the remaining three regions. In the stratiform precipitation, the radar reflectivity CFADs of four regions clearly show enhanced values around 4 − 5 km, which signifies the presence of a well-defined bright band caused by the melting of ice particles while crossing the zero degrees isotherm level^18,19,47^. Above the melting layer, a higher (> 10.5%) occurrence frequency of radar reflectivity between 20 − 25 dBZ is predominant in north Taiwan. Such higher frequency is shabby in central and east Taiwan and mostly absent in south Taiwan. Different from the higher frequency of lower reflectivity values in north Taiwan, south Taiwan shows dominant frequency (7.5–10.5%) at radar reflectivity values ranging between ~ 28 − 32 dBZ. For the mass-weighted diameter value of 1 mm, the occurrence frequency greater than 10.5% strongly persists in north Taiwan, followed in east and central Taiwan. Despite that, drops of *D*_*m*_ values 1.5 − 2 mm are more dominant in central Taiwan's warm rain regions (< 5 km). The normalized intercept parameter occurrence frequency of 1.5% and above exhibits broader and deeper distribution in south and central Taiwan than the north and east Taiwan.

**Figure 7:**
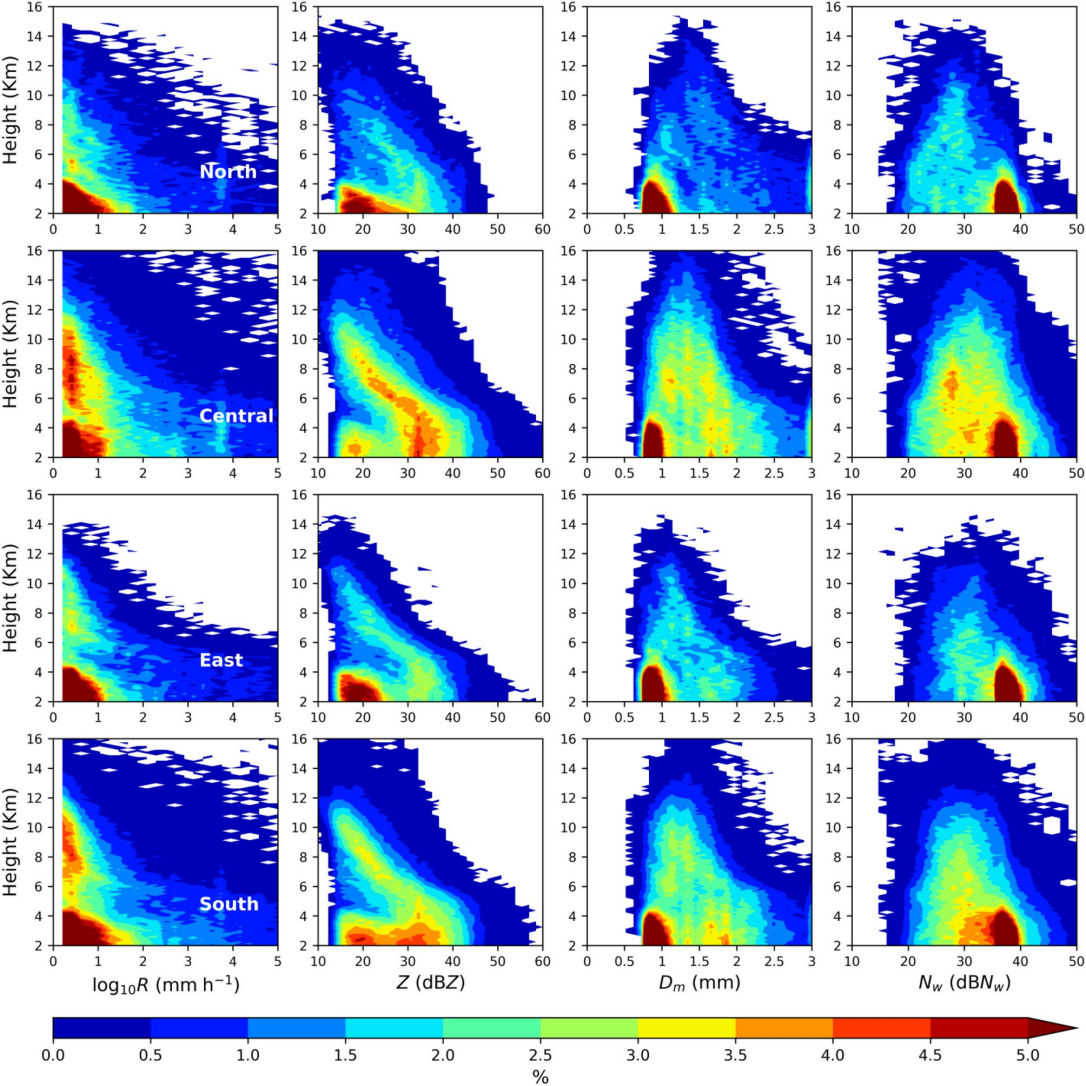
Contour frequency by altitude diameter (CFAD) of rainfall rate (*R*, mm h^−1^) (first column), radar reflectivity (*Z*, dBZ) (second column), mass-weighted mean diameter (*D*_*m*_, mm) (third column), and normalized intercept parameter (dB*N*_*w*_: 10log_*10*_*N*_*w*_, *N*_*w*_ in m^−3^ mm^−1^) (fourth column) for the summer season *convective rainfall* over north (first row), central (second row), east (third row) and south (fourth row) Taiwan.

**Figure 8:**
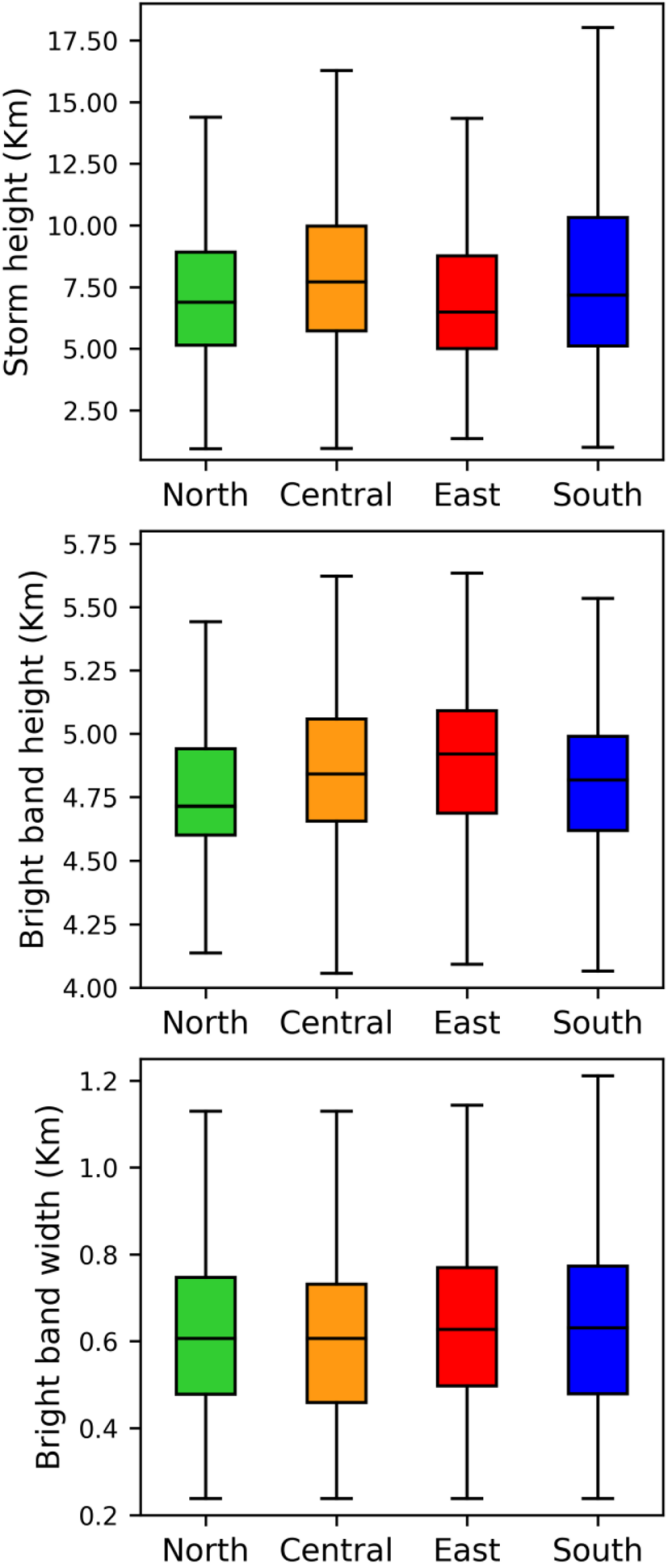
Storm heights (top row), bright band heights (middle row), and bright band widths (bottom row) of summer season precipitating clouds over north, central, east and south Taiwan.

Contrary to the CFADs of the RSD parameters (log_10_*R*, *Z*, *D*_*m*_, and 10log_10_*N*_*w*_) in stratiform precipitation, these parameters in the convective precipitation display higher values with broader and deeper distributions (Fig. 7). From the standpoint of the four regions of Taiwan, the echo tops of convective precipitations are relatively higher than the stratiform precipitation. More particularly, the echo tops in central and south Taiwan were above 16 km in convective precipitation and below 16 km in stratiform precipitation. In addition, the near-surface maximum reflectivity values are greater (smaller) than 50 dBZ in convective (stratiform) precipitation. Furthermore, for the given precipitation (stratiform or convective) type, the echo tops in central and south Taiwan are relatively higher than the north and east Taiwan (Fig. 8). The extent of higher occurrence frequency of rainfall rates to deeper altitudes (above the melting layers heights) in central and south Taiwan infers the predominance of deep convective clouds in these two regions; besides, such higher occurrence frequency of rainfall rates are mostly confined to below melting layers heights (less than 5 km) in the eastern and northern Taiwan Taiwan signifying the prevalence of shallow convection over north and east Taiwan. While moving from 10 to 5 km altitude, the radar reflectivity CFADs of four regions show an evident increase in occurrence frequency with the increase in radar reflectivity and a slight decrease from 5 km to the near-surface. The rise in *Z* and *D*_*m*_ values from 10 to 5 km altitude can be correlated to the growth of ice particles by riming and aggregation while they descend^11,48,49^. Below 4 km height, the radar reflectivity shows double peak distributions around 20 and 35 dBZ over the four regions of Taiwan. Following the radar reflectivity, the *D*_*m*_ also hinted a double peak distribution around 0.8 and 1.8 mm in the warm rain region (< 4 km). Such double peak distributions in the warm rain region (around *D*_*m*_ values of 1 & 2 mm) were also reported by Chen, et al.^11^ for the summer monsoon convective precipitation over China’s Yangtze–Huaihe River Valley. They attributed such peak distributions (at lower reflectivity or *D*_*m*_ values) to the dominance of light convection with a higher concentration of small-size precipitating particles. However, the second peak frequency in central and south Taiwan is located at relatively higher reflectivity values (~ 30 − 38 dBZ for central Taiwan and ~ 25 − 35 dBZ in south Taiwan) than the north and east Taiwan. Below the melting layer (< 5 km), the smaller drops (*D*_*m*_ < 1 mm) exhibit higher occurrence frequency in four regions of Taiwan; on the other hand, the occurrence frequency of mid-size (1 mm < *D*_*m*_ < 2 mm) and large (*D*_*m*_ > 2 mm) drops are more in the central and south Taiwan, and the frequency of large size drops is < 1% in north Taiwan. Nonetheless, between the central and south Taiwan convective precipitation, 1.5 − 4% occurrence frequency persists to deeper levels (above 10 km) in central Taiwan than in south Taiwan. From the CFADs of *D*_*m*_ and dB*N*_*w*_, it can be inferred that the higher concentration of mid and large-size ice and liquid particles are predominant above and below the melting layers heights of central Taiwan convective precipitation.

### Mean profiles of rain and RSD parameters

**Figure 9:**
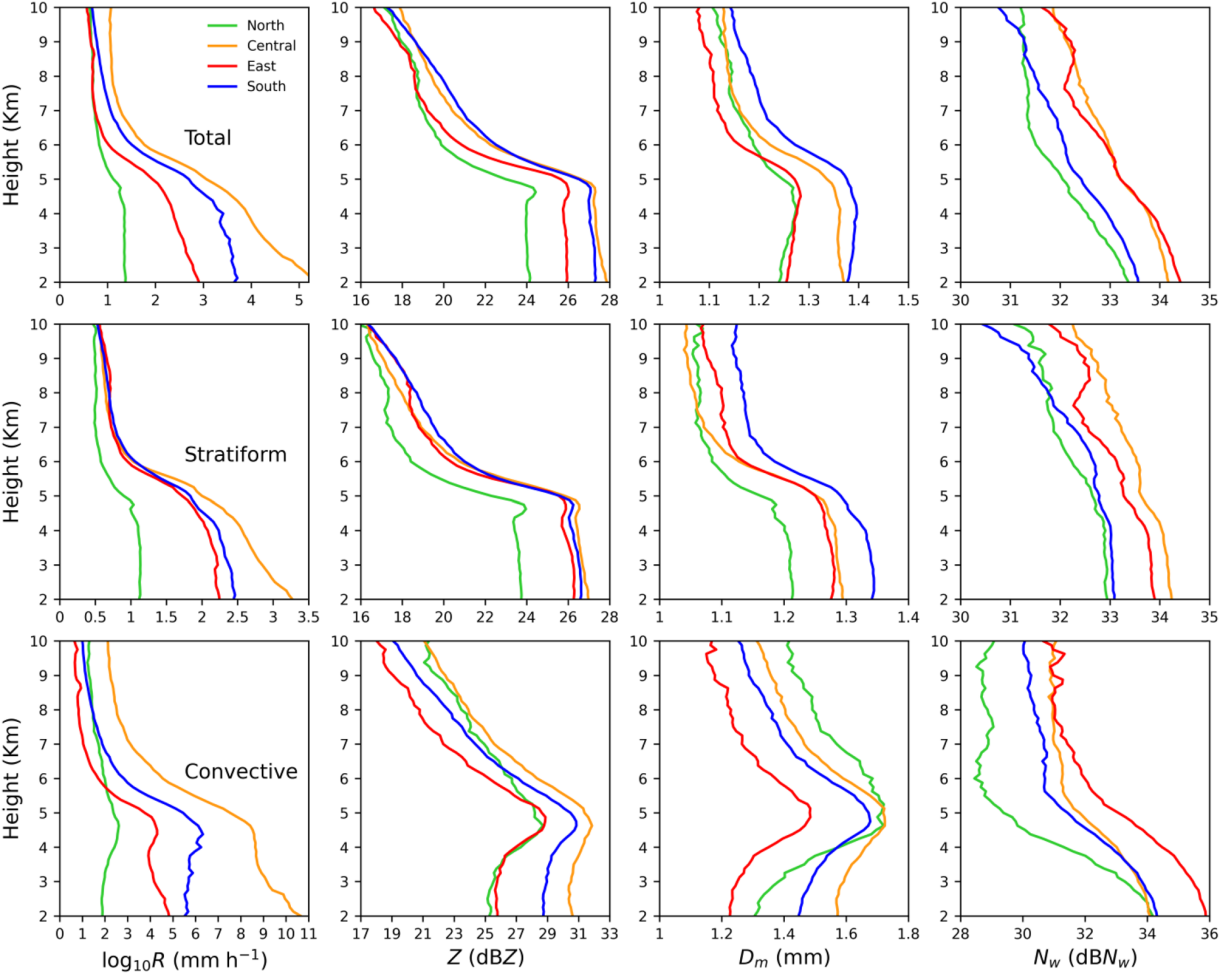
Mean profiles of rainfall rate (*R*, mm h^−1^) (first column), radar reflectivity (*Z*, dBZ) (second column), mass-weighted mean diameter (*D*_*m*_, mm) (third column), and normalized intercept parameter (dB*N*_*w*_: 10log_*10*_*N*_*w*_, *N*_*w*_ in m^−3^ mm^−1^) (fourth column) for the total (first row), stratiform (second row), and convective precipitations (third row) of summer season over north (green), central (orange), east (red) and south (blue) Taiwan.

To further delineate the RSD features and the underlying microphysics process among the four regions of Taiwan, the mean profiles of rainfall rate, radar reflectivity, mass-weighted mean diameter, and normalized intercept parameter are exemplified in Fig. 9. A gradual increase in mean rainfall rate, radar reflectivity, and mass-weighted mean diameter from 10 to 05 km is noticeable for four regions of Taiwan. However, this increasing tendency is more prominent in convective precipitation suggesting the growth of liquid and ice particles to a large size in convective precipitation. On the contrary, below the melting layer (below 5 km height), a decrease (a slight increase or no change) in the mean reflectivity and *D*_*m*_ values can be seen in the convective (stratiform) precipitation. Besides, the drop concentration profiles (dB*N*_*w*_) of stratiform precipitation reveal not much variation with the descending altitude, especially in the warm rain regions, whereas, in the case of convective rainfall, an increase in the mean number concentration is evident. A decrease in drop size and increase in number concentration in the warm rain regions (below 4 km height) hints at the dominance of collision-breakup processes in the convective precipitation; on top of that, a slight increase in drop size with not much variation in the number concentration bring up the equilibrium condition among collision-coalescence and breakup processes in the stratiform precipitation. Aside from the disparities in the microphysical processes between the stratiform and convective rains, for the given precipitation (total, stratiform, or convective) type, such deviations are also apparent among the four regions of Taiwan. The total precipitation mean rainfall rate and radar reflectivity profiles confirm larger values in central and south Taiwan, followed in east and north Taiwan. Such higher rainfall rates in the total precipitations of central and south Taiwan, which can be inferred from the mean profiles of drop size (*D*_*m*_) and number concentration (dB*N*_*w*_), are ascribed to the governance of more concentration of large drops.

### Microphysical characteristics in the warm rain region

**Figure 10:**
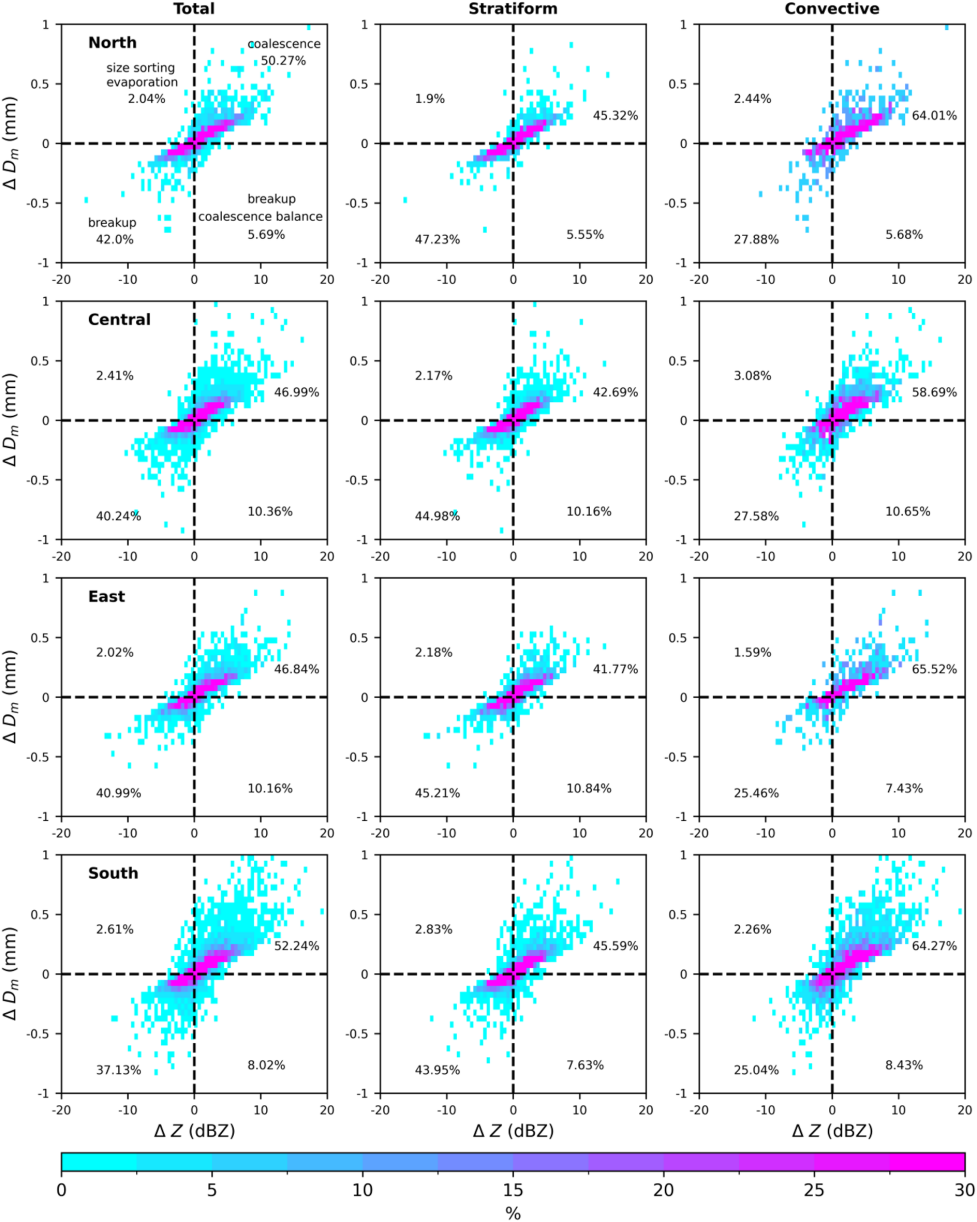
Probability density distributions of mass-weighted mean diameter and radar reflectivity in the 1 km layer depth (Δ*D*_*m*_ = (*D*_*m*_)_2 km_ − (*D*_*m*_)_3 km_ and Δ*Z* = (*Z*)_2 km_ − (*Z*)_3 km_) in the warm rain regions of summer clouds over north (first row), central (second row), east (third row), and south (fourth row) Taiwan for total (first column), stratiform (second column), and convective (third column) precipitation.

While exploring the warm rain microphysical processes (collision-coalescence, breakup, and evaporation), previous researchers adopted the combined information of radar reflectivity and differential radar reflectivity over a 3 km layer depth from 3.5 km to 0.5 km^50^. Owing to the unavailability of differential radar reflectivity in the GPM DPR and the direct relation of differential reflectivity with the mass-weighted mean diameter, the GPM DPR *Z* and *D*_*m*_ values below the melting layer are used to understand the warm rain microphysical process^13,49,51,52^. Considering the susceptibility of the warm rain region on zero degrees isotherm, which varies with the geographic area, different height levels for the warm layer depth were adopted in the literature^13,49,51^. Since the melting layers in summer over Taiwan are well above the 3 km height level (Fig. 8)^18,19^, the present study adopted the warm layer depth between the height levels of 2 to 3 km. Figure 10 illustrates the probability density distributions estimated for radar reflectivity and mass-weighted mean diameter values of the 1 km layer depth for the total, stratiform and convective precipitations of four regions. An increase in mass-weighted mean diameter and radar reflectivity can be related to the coalescence process and the decrease in Z and increase in *D*_*m*_ to size-sorting or evaporation (which reduces the number of small droplets). On the other hand, an increase in Z and a slight decrease in mass-weighted mean diameter infer the stability between the breakup and coalescence process. Figure 10 shows that, among the four regions of Taiwan and two precipitation types, the coalescence and breakup processes are more dominant than the size sorting/evaporation and breakup-coalescence balance. However, for the given region of Taiwan (north/central/south/east Taiwan), the stratiform precipitations prevail with breakup processes and the convective rains with the coalescence processes.

### Discussion

Investigation of the microphysical attributes of summer season rainfall using long-term GPM DPR rain/RSD parameters demonstrated substantial dissimilarities among four regions of Taiwan. The shooting of summer season precipitation clouds to deeper altitudes in south and central Taiwan than the east and north Taiwan can be attributed to the interaction of southwesterly monsoon flow with the CMR. Moreover, central Taiwan's CFAD of echo top heights (Figs. 5, 6 and 7) and median values of storm heights (Fig. 8) reveal higher values than south Taiwan. On the other hand, north Taiwan showed relatively lower storm and echo top heights than the south and cenral Taiwan (Figs. 5, 6, 7 and 8), which hits the association of predominant shallow cloud systems and fewer deeper cloud systems. The growth of ice particles by riming and aggregation processes is significant in south and central Taiwan, more particularly predominant in south Taiwan. Considerable growth of ice particles in south Taiwan is related to the abundant stratiform precipitation (Fig. 6) with wider bright band widths (Fig. 8). In contrast, for central Taiwan, predominant ice particle growth is caused by the deep convective precipitation (Fig. 7) with enhanced echo tops (Fig. 8). In the warm rain regions of stratiform precipitation, larger *D*_*m*_ smaller *N*_*w*_ values in south Taiwan than in central Taiwan (Fig. 9) can be related to the predominance of size sorting-evaporation and coalescence processes in south Taiwan; breakup and breakup-coalescence balance in central Taiwan (Fig. 10). Relatively higher bright band heights in east Taiwan than in north Taiwan (Fig. 8) provide adequate time for the raindrops to attain equilibrium among collision-coalescence and breakup processes. Hence, larger *D*_*m*_ and *N*_*w*_ values in the stratiform precipitation of east Taiwan than in north Taiwan can be associated with the predominance of collision-coalescence balance and size sorting-evaporation processes in east Taiwan. In the warm rain regions of convective precipitation, a balance among collision-coalescence and breakup processes in east Taiwan and enhanced breakup, size sorting-evaporation process in north Taiwan (Fig. 10) resulted in smaller *D*_*m*_, larger *N*_*w*_ values in east Taiwan than in north Taiwan (Fig. 9). Further, an enhanced collision-coalescence process in south Taiwan, size sorting-evaporation, and a balance among collision-coalescence and breakup processes in central Taiwan (Fig. 10) resulted in larger *D*_*m*_ and slightly smaller *N*_*w*_ values in central Taiwan than in south Taiwan (Fig. 9).

## Summary

The complex topography of Taiwan can serve as a natural test bed to understand the interaction of summer monsoon precipitation clouds with its terrain and the underlying microphysical processes. Hence, for the first time, regional discrepancies in the microphysical characteristics of summer season rainfall over Taiwan are investigated using long-term (2014–2022) observations of GPM DPR. In this study, near-surface precipitation and particle size parameters like rainfall rate, radar reflectivity, mass-weighted mean diameter, normalized intercept parameters, and their vertical profiles from level 2 data product of GPM DPR (2ADPR, version 7) over Taiwan are used. The summer season rainfall characteristics over the four regions (that are segregated with reference to central mountain ranges) of Taiwan demonstrate apparent dissimilarities. From the contoured frequency by altitude diagrams (CFADs) of radar reflectivity, rainfall rate, and storm heights information, it can be inferred that the summer monsoon clouds over the south and central region are extended to deeper altitudes. The deeper extent of precipitating clouds over south and central Taiwan can be attributed to the blocking and uplifting of southwesterly monsoon clouds by the central mountain ranges. The deeper convection in central and southern Taiwan aids in the growth of ice/supercooled liquid particles (in the cold regions) and liquid particles (in the warm regions) to relatively larger sizes than the north and east Taiwan, and it is confirmed by the CFADs of particle size (*D*_*m*_) and number concentration (log10*N*_*w*_) information. The convective clouds of relatively lower storm heights produce a higher concentration of small-size drops in north Taiwan. Alternatively, the dominance of higher concentrations of bigger drops over central and south Taiwan leads to higher rainfall amounts in central and south Taiwan. The present study reveals the dominance of size sorting-evaporation processes in the south and central Taiwan's total, stratiform, and convective precipitations; collision-coalescence process in north and south Taiwan's total and stratiform precipitation and in east and south Taiwan's convective rainfall. The balance among collision-coalescence and breakup processes is predominant in central and east Taiwan’s total and stratiform precipitation and in central and south Taiwan’s convective precipitation. The breakup process is dominant in north and east Taiwan's total and stratiform precipitation and in central and north Taiwan's convective precipitation.

## Data Availability

The GPM DPR data is provided by the NASA Goddard Space Flight Center′s Mesoscale Atmospheric Processes Laboratory and Precipitation Processing System (PPS) can be downloaded from https://pmm.nasa.gov/dataaccess/downloads/gpm.
